# Inhibitory Effect of Gamma-Irradiated Chitosan on the Growth of Denitrifiers

**DOI:** 10.1155/2009/418595

**Published:** 2010-02-17

**Authors:** Javier Vilcáez, Tomohide Watanabe

**Affiliations:** ^1^New Industry Creation Hatchery Center (NICHe), Tohoku University, Aoba 6-6-20, Aramaki, Aoba-ku, Sendai, Miyagi 980-8579, Japan; ^2^Graduate School of Engineering, Gunma University, 1-5-1 Tenjin-cho, Kiryu, Gunma 376-8515, Japan

## Abstract

In order to find an environmentally benign substitute to hazardous inhibitory agents, the inhibitory effect of *γ*-irradiated chitosans against a mixed culture of denitrifying bacteria was experimentally evaluated. Unlike other studies using pure aerobic cultures, the observed effect was not a complete inhibition but a transient inhibition reflected by prolonged lag phases and reduced growth rates. Raw chitosan under acid conditions (pH 6.3) exerted the strongest inhibition followed by the 100 kGy and 500 kGy irradiated chitosans, respectively. Therefore, because the molecular weight of chitosan decreases with the degree of *γ*-irradiation, the inhibitory properties of chitosan due to its high molecular weight were more relevant than the inhibitory properties gained due to the modification of the surface charge and/or chemical structure by *γ*-irradiation. High dosage of *γ*-irradiated appeared to increase the growth of mixed denitrifying bacteria in acid pH media. However, in neutral pH media, high dosage of *γ*-irradiation appeared to enhance the inhibitory effect of chitosan.

## 1. Introduction

Chitosan is known to be biodegradable, biocompatible, nontoxic, nonallergenic, renewable material. Chitosan is produced commercially by deacetylation of chitin which is the second most abundant polymer after cellulose. Due to its unique polycationic nature, chitosan has been shown to possess antibacterial as well as inhibitory properties against a wide variety of bacteria and fungi [1, 2]. Therefore, from an environmental point of view, chitosan appears to be an excellent candidate to replace hazardous antibacterial agents such as chlorine, nitrite, glutaraldehyde and quaternary ammonium derivatives. However, the bottleneck of the application of chitosan is its low solubility in neutral pH media [3]. In this respect, recent reports have shown that *γ*-irradiation, depending on the irradiation dose, produced chitosans of lower molecular weight with different surface charge and even different chemical structure which not only increased the solubility of chitosan in water [4] but also improved its inhibitory effect [5]. It is generally agreed that the inhibitory effect of chitosan is influenced by many factors such as its molecular weight, concentration in solution, degree of deacetylation, relative surface charge and pH of the media [2]. Depending on the degree of irradiation, the same variables appear to influence the inhibitory effect of *γ*-irradiated chitosan products [5].

Regarding the efficiency of *γ*-irradiated products, it has been reported that 125 mg/L of chitosan irradiated with 100 kGy was effective in inhibiting the growth of *E. coli* completely [5]. Although mixed cultures are more frequent in practical situations, no evidence has been provided to demonstrate that *γ*-irradiated chitosan products are also effective in inhibiting the growth of other species and/or mixed cultures. Therefore, in this study, a mixed culture of anoxic denitrifying bacteria was used in order to evaluate the inhibitory effect of *γ*-irradiated chitosan products. 

Two 100% deacetylated chitosans, one irradiated with 100 kGy and the other irradiated with 500 kGy in air medium, were evaluated together with a raw 100% deacetylated chitosan product. The 100% deacetylated chitosan product was used because the inhibitory effect of chitosan has been reported to increase with the degree of deacetylation [1].

## 2. Material and Methods

At first, an inoculum of mixed denitrifying bacteria, harvested from a 40 L denitrifying reactor operated in our laboratory was subcultured in a 2.5 L Erlenmeyer flask at 30°C and a constant pH of 7.4. Fill-and-draw operations where well mixed 0.5 L aliquots were periodically replaced by fresh cultivation medium allowed us to be confident that the inoculums were in the exponential growth phase. The cultivation medium used in this study contained KNO_3_, 4.3 g/L; CH_3_COONa·3H_2_O, 6.85 g/L; NaHCO_3_, 3.3 g/L; KH_2_PO_4_, 0.05 g/L; K_2_HPO_4_, 0.04 g/L; NaCl, 0.02 g/L; CaCl_2_·2H_2_O, 0.025 g/L; MgSO_4_·7H_2_O, 0.08 g/L; FeCl_3_·6H_2_O, 0.04 g/L; and NaMo_4_, 0.001 g/L. Acetate was used as the sole carbon source. 

Stock solutions of the *γ*-irradiated and raw chitosans were prepared by dissolving 0.2 g in 10 mL autoclaved distilled water containing 5% acetic acid solution.

Sterilized vials were filled with 100 mL of the cultivation medium. Then, inoculums at the exponential growth phase collected from the culture vessel were inoculated such that the desired initial concentration of denitrifying bacteria was obtained in each vial. 

Right after inoculation of the vials, the stock solution of chitosan was dosed to each vial up to the desired concentration. The initial pH in the vials was adjusted to 6.3 by the addition of acetic acid or 0.1 M sodium hydroxide solution. 

After stripping with nitrogen gas for 30 minutes, the vials were capped with rubber caps and placed in reciprocating shaker at 30°C. The concentrations of nitrate and nitrite as well as the number of live denitrifying bacteria were monitored. Each vial test was done in triplicate. A blank test of only cultivation media and denitrifiers accompanied every test.

The plate counting method was used to determine the number of live bacteria. The medium in the agar plates contained: Difco Bacton yeast extract, 5 g/L; KH_2_PO_4_, 0.1 g/L; KNO_3_, 1 g/L; and Agar, 25 g/L. 0.1 mL aliquots of selected dilutions of samples were uniformly spread on the solid agar. The plates were anoxically incubated for 4 days in an incubator at 30°C. Nitrate and nitrite were measured by ion chromatography (Yokogawa Analytical Systems, IP-7000).

## 3. Results

The growth curves were clearly divided into the lag and exponential growth phases. Therefore, in this study, two indicators were used to evaluate the inhibitory effect of the chitosans. 

The ratio of the lag phase duration of the cultures treated with chitosan (Δ*t*_Treated  sample_) to that of the blank culture without chitosan (Δ*t*_Blank  sample_) was used as one of the indicators: IF_Lag  phase_ = Δ*t*_Treated  sample_/Δ*t*_Blank  sample._

The ratio of the specific consumption rate of the cultures treated with chitosan (*μ*_Treated  sample_) to that of the blank culture without chitosan (*μ*_Blank  sample_) was used as the other inhibitory indicator: IF_Exponential  phase_ = *μ*_Treated  sample_/*μ*_Blank  sample_. The specific nitrate consumption rates were calculated assuming a first order Monod type kinetics. 

### 3.1. Effect of the Concentration of the Raw and *γ*-Irradiated Chitosans

**Figure 1:**
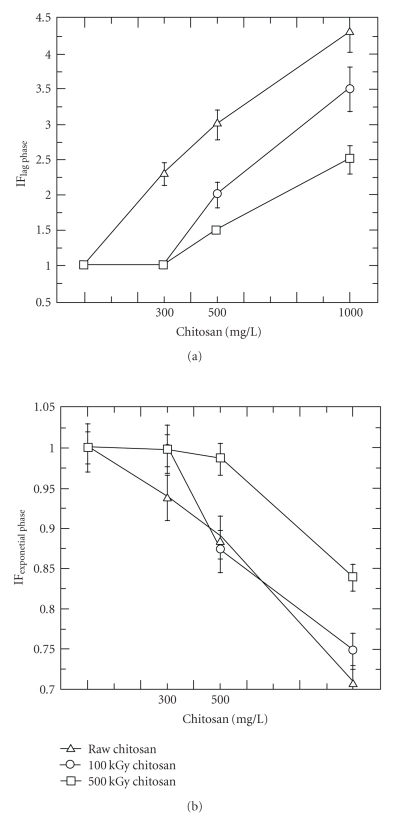
Effect of the concentration of raw and *γ*-irradiated chitosans on the duration of the lag phase (a) and on the specific consumption rates of denitrifiers at the exponential phase (b) (Δ*t*_Blank  sample_ = 24 h; *μ*_Blank  sample_ = 0.57 h^−1^; [CFU/mL]_initial_ = 5 × 10^5^; initial pH = 6.3).

**Figure 2:**
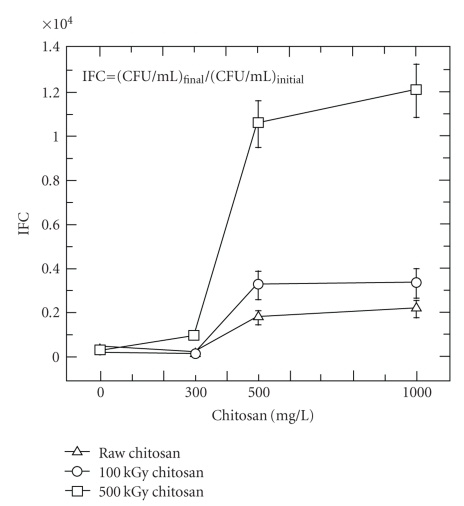
Effect of the concentration of raw and **γ*-*irradiated chitosans on the concentration of denitrifiers after complete nitrate consumption ([CFU/mL]_initial_ = 5 × 10^5^ CFU/mL; initial pH = 6.3).

Figures 1 and 2 show the response of the denitrifying bacteria to different concentrations of 100 kGy irradiated, 500 kGy irradiated, and raw chitosans. 

Figure 1 shows that the raw chitosan requires a lower minimum effective concentration than both 100 kGy and 500 kGy irradiated chitosans. While the application of 300 mg/L of raw chitosan sufficed to inhibit the growth of denitrifiers in the lag phase, the 100 kGy and 500 kGy irradiated chitosans required an application of 500 mg/L. The duration of the inhibition increased with increasing the concentration of the raw and *γ*-irradiated chitosans. However, unexpectedly, the duration of the inhibition decreased with decreasing the degree of irradiation, the raw chitosan exerted longest inhibition followed by the 100 kGy irradiated chitosan and the 500 kGy irradiated chitosan respectively. A momentary reduction on the number of live bacteria of one order of magnitude, from 5 × 10^5^ to 5 × 10^4^ CFU/mL, was detected for 1000 mg/L of the raw and 100 kGy irradiated chitosan chitosans. 

Figure 1 also shows the effect of the concentration of the raw and *γ*-irradiated chitosans on the specific consumption rate of denitrifying bacteria at the exponential growth phase. In the same way as in the lag phase, the raw chitosan showed a stronger capacity for inhibition as well as a lower minimum effective concentration (300 mg/L) than both *γ*-irradiated chitosans. Comparing the performance of both *γ*-irradiated chitosans during the exponential phase, the 100 kGy irradiated chitosan had higher inhibition effect than that of the 500 kGy irradiated chitosan.

Figure 2 shows differences in the number of live denitrifying bacteria after complete consumption of nitrate. The final number of live denitrifying bacteria increased with an increase in the dosed concentration of chitosan. Since the initial total concentration of acetate was the same in all vial tests, chitosan might have served as an additional source of carbon and energy for the proliferation of denitrifiers. If this is the case, according to the ratio of final to initial CFU/mL (*IFC*), the 500 kGy irradiated chitosan is more readily metabolized than the 100 kGy irradiated chitosan which in turn is more readily metabolized than the raw chitosan. The fact that **γ**-irradiation has a strong effect on the molecular weight of chitosan suggests that the observed behavior might be closely related to the lower molecular weight acquired due to **γ**-irradiation.

### 3.2. Effect of pH

**Figure 3:**
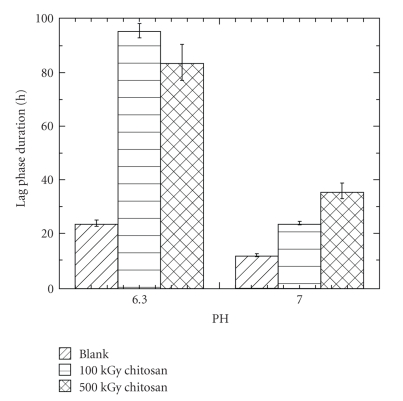
Effect of the pH on the duration of the lag phase in media containing 1000 mg/L of *γ*-irradiated chitosans ([CFU/mL]_initial_ = 5 × 10^5^).

Figure 3 shows the effect of the pH on the duration of the lag phases of denitrifiers in media containing 1000 mg/L of *γ*-irradiated chitosans at pH 6.3 and pH 7.0. Regardless of the longer lag phase at pH 6.3 than at pH 7.0 in the blank cultures without chitosans, the inhibition caused by both *γ*-irradiated chitosans at pH 6.3 is much longer than at pH 7.0. This result demonstrates the dependence of the *γ*-irradiated chitosans on the pH of the medium. On the other hand, it is noteworthy that the inhibition effect of the *γ*-irradiated chitosans at pH 7.0 is reduced but not completely suppressed. What is more, at neutral pH conditions the inhibition caused by the 500 kGy irradiated chitosan is stronger than the inhibition caused by the 100 kGy irradiated chitosan, suggesting that at neutral pH conditions, the inhibitory effect of *γ*-irradiated chitosans increased with the degree of *γ*-irradiation, probably because of the higher solubility achieved due to the lower molecular weight that result from the *γ*-irradiation.

**Figure 4:**
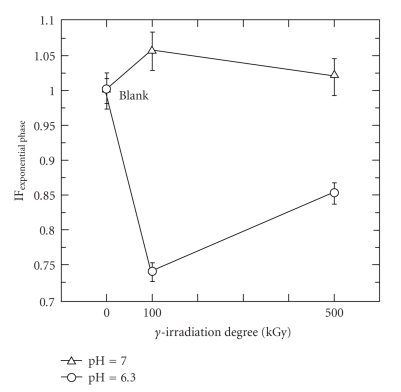
Effect of pH on specific consumption rate in media containing 1000 mg/L of **γ**-irradiated chitosan ([CFU/mL]_initial_ = 5 × 10^5^; *μ*_Blank  sample_ = 0.57 h^−1^).

During the exponential phase, as observed in Figure 4, when the sample contains *γ*-irradiated chitosans at neutral pH conditions, the specific consumption rates remained practically constant regardless of the different chitosans. This indicated that during the exponential phase, neutral pH conditions suppressed the inhibition capacity of both **γ**-irradiated chitosans. 

Although the solubility of raw and gamma-irradiated chitosans was not quantified in this study, the difference in the solubility of these two types of chitosans was evident. While a homogeneous solution was observed with the gamma-irradiated chitosans, the raw chitosan formed precipitates at neutral pH conditions. Therefore, the performance of the raw chitosan at neutral pH conditions could not be evaluated.

### 3.3. Effect of the Initial Concentration of Denitrifiers

**Figure 5:**
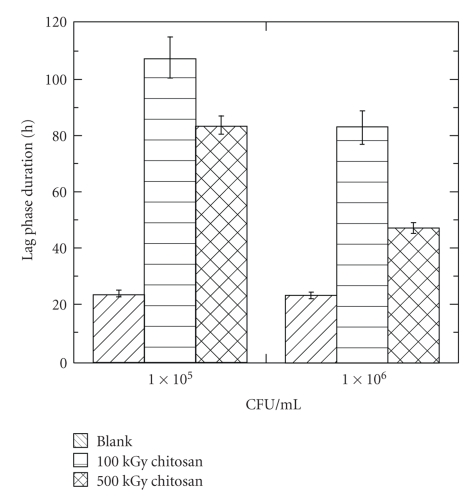
Effect of the initial concentration of denitrifiers on the duration of the lag phase in media containing 1000 mg/L of **γ*-*irradiated chitosans (Initial pH = 6.3).

Figure 5 shows that the duration of the inhibition decreased with an increase in the initial concentration of denitrifiers. This suggested that there is a specific chitosan dose such as mg-chitosan/mg-bacteria that should be used as operational parameter for an effective inhibitory performance.

**Figure 6:**
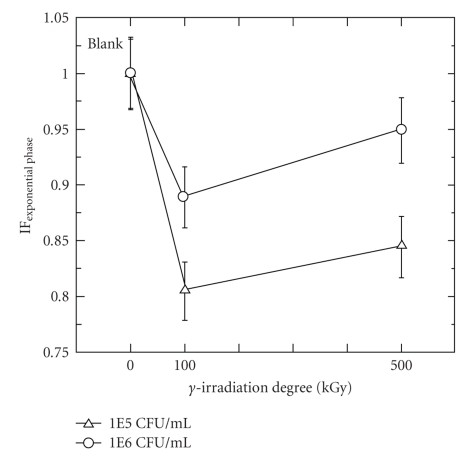
Effect of the initial concentration of denitrifiers on the specific rate coefficient in media containing 1000 mg/L of *γ*-irradiated chitosans (*μ*_Blank  sample_ = 0.57 h^−1^. Initial pH = 6.3).

Figure 6 shows the specific consumption rate at different initial concentration of denitrifiers. The specific consumption rates increase with an increase in the bacterial concentration regardless of the degree of **γ*-*irradiation. The 500 kGy irradiated chitosan was less resistant to an increment on the initial concentration of denitrifiers than the 100 kGy irradiated chitosan.

## 4. Discussion

The better inhibitory performance of the raw chitosans, which have higher molecular weights than *γ*-irradiated chitosans, is in accordance with the results reported by Shin et al. [2] where chitosans of high molecular weights was found to be more effective in inhibiting the growth of aerobic bacteria than chitosans of low molecular weights. On the other hand, the results obtained in this investigation differed from the results reported by Matsuhashi and Kume [5], where 125 mg/L of 100 kGy irradiated chitosan was found to completely inhibit the growth of aerobic bacteria. Therefore, similar to the case where the effect of molecular weight of chitosan on the inhibition efficiency was found to depend on the species of the bacteria [1, 6, 7], the effect of the degree of *γ*-irradiation on the inhibitory capacity of chitosan appears to depend on the species of the bacteria too. 

The effect of *γ*-irradiation on the molecular weight of chitosan has been extensively studied. For instance, Duy Lan and Gang Diep have reported that the average molecular weight of a 90% deacetylated chitosan is reduced from 560,000 Da to 139000, 112000 and 72500 Da when irradiated at 40, 75 and 100 kGy in air medium, respectively [8]. In another study, it has been reported that a dose of 300 kGy in air medium reduces the average molecular weight of chitosan from 690000 to 20900 Da [9]. On the basis of this information, it can be inferred that the 500 kGy irradiated chitosan of this research had much lower molecular weight than the 100 kGy and 300 kGy irradiated chitosans, and that this low molecular weight could have enhanced its solubility and inhibitory effect at pH 7.0. 

Ratajska et al. have shown that chitosans with low average molecular weight (e.g., 97000 Da) were more readily metabolized by microorganisms present in active sludge [10]. Since denitrifiers are usually present in active sludge, and *γ*-irradiation considerably reduces the molecular weight of chitosan, most probably, the increased final live bacteria numbers was due to the metabolization of the raw and *γ*-irradiated chitosans.

In summary, the effect was not complete inhibition, as reported for pure aerobic cultures, but a partial inhibition reflected by prolonged lag phases and slower growth rates.

## 5. Conclusions

The raw 100% deacetylated chitosan has shown better inhibition properties against mixed cultures of denitrifying bacteria than the 100 kGy and 500 kGy irradiated chitosans. In contrast to the results under acid medium conditions, the inhibitory effect of the 500 kGy irradiated chitosan, under neutral pH conditions, was higher than the inhibitory effect of the 100 kGy irradiated chitosan, suggesting that *γ*-irradiation dosages higher than 500 kGy might increase the inhibitory effect of chitosan at neutral pH conditions. However, according to the specific consumption rates and final live bacteria numbers, there might be a maximum threshold degree of *γ*-irradiation beyond which instead of improving the inhibition activity of chitosan *γ*-irradiation might facilitate the growth of denitrifiers.
